# Sildenafil Reduces Insulin-Resistance in Human Endothelial Cells

**DOI:** 10.1371/journal.pone.0014542

**Published:** 2011-01-28

**Authors:** Caterina Mammi, Donatella Pastore, Marco F. Lombardo, Francesca Ferrelli, Massimiliano Caprio, Claudia Consoli, Manfredi Tesauro, Lucia Gatta, Massimo Fini, Massimo Federici, Paolo Sbraccia, Giulia Donadel, Alfonso Bellia, Giuseppe M. Rosano, Andrea Fabbri, Davide Lauro

**Affiliations:** 1 Centre for Clinical and Basic Research, IRCCS San Raffaele Pisana, Rome, Italy; 2 Department of Internal Medicine, University of Tor Vergata, Rome, Italy; 3 San Raffaele Sulmona, L'Aquila, Italy; 4 Unit of Endocrinology, Department of Internal Medicine, Ospedale S. Eugenio and CTO A. Alesini, University of Tor Vergata, Rome, Italy; Istituto Dermopatico dell'Immacolata, Italy

## Abstract

**Background:**

The efficacy of Phosphodiesterase 5 (PDE5) inhibitors to re-establish endothelial function is reduced in diabetic patients. Recent evidences suggest that therapy with PDE5 inhibitors, i.e. sildenafil, may increase the expression of nitric oxide synthase (NOS) proteins in the heart and cardiomyocytes. In this study we analyzed the effect of sildenafil on endothelial cells in insulin resistance conditions *in vitro*.

**Methodology/Principal Findings:**

Human umbilical vein endothelial cells (HUVECs) were treated with insulin in presence of glucose 30 mM (HG) and glucosamine 10 mM (Gluc-N) with or without sildenafil. Insulin increased the expression of PDE5 and eNOS mRNA assayed by Real time-PCR. Cytofluorimetric analysis showed that sildenafil significantly increased NO production in basal condition. This effect was partially inhibited by the PI3K inhibitor LY 294002 and completely inhibited by the NOS inhibitor L-NAME. Akt-1 and eNOS activation was reduced in conditions mimicking insulin resistance and completely restored by sildenafil treatment. Conversely sildenafil treatment can counteract this noxious effect by increasing NO production through eNOS activation and reducing oxidative stress induced by hyperglycaemia and glucosamine.

**Conclusions/Significance:**

These data indicate that sildenafil might improve NOS activity of endothelial cells in insulin resistance conditions and suggest the potential therapeutic use of sildenafil for improving vascular function in diabetic patients.

## Introduction

Nitric oxide (NO) is a reactive free radical gas acting as an intracellular or extracellular messenger. It is synthesized from L-arginine by a family of isoforms of NO synthases (NOS 1–3). NO generation by NOS-3 (eNOS) in endothelial cells explains the effects of endothelial-dependent vasodilators on vascular relaxation and decreased platelet adhesion and aggregation. These and others effects of NO are mediated by increased cyclic GMP (c-GMP) formation due to soluble guanylyl cyclase activation.

In turn c-GMP activates protein kinase G (PKG) and through the phosphorylation of its substrates can regulate many other biological processes. The increase of c-GMP is counteracted by Phosphodiesterase 5 (PDE5) activity that transformes c-GMP into GMP.

e-NOS activity is regulated by agonist-induced elevation of intracellular free Ca^2+^ concentration with subsequent binding of Ca^2+^/Calmodulin (Ca^2+^/CaM) to e-NOS. However shear stress and isometric contraction have been shown to activate the enzyme in a Ca^2+^/CaM-independent manner [1], through the activation of c-GMP dependent PKG [2]. This mechanism may serve to ensure continuous production of NO, independently of fluctuations in intracellular Ca^2+^ levels. Although eNOS was initially reported to be phosphorylated exclusively on serine residues [3] other evidence has shown that eNOS is also phosphorylated in threonine- and tyrosine-domains [4]. NO production in endothelial cells is regulated by phosphorylation of several eNOS consensus sequence sites by means of protein kinase (PK) Akt-1, PKC and calmodulin kinase II [5].

In addition to its well known metabolic action, insulin has vasodilatory effects that depend on NO production in the vascular endothelium. It has been demonstrated that e-NOS activation induced by insulin is mediated by the PI3K-Akt pathway resulting in e-NOS phosphorylation in Ser^1177^ independently of Ca^2+^-CaM pathway [6]. Moreover insulin inhibition of PI3K/Akt-1 pathway leads to impaired NO availability [7], [8]. Reduced insulin-mediated vasodilatation might contribute to vascular damage in insulin-resistant states. We already demonstrated that hyperglycemia, via hexosamine pathway activation, induces selective insulin resistance in endothelial cells and impairs e-NOS activation [9].

Sildenafil citrate is a PDE5 inhibitor (PDE-5i) that belongs to the “new class” of PDE-5i. PDE-5 selectively degrades cyclic guanosine monophosphate (cGMP), hence its inhibition by sildenafil raises cGMP intracellular levels inducing vasodilatation. In recent years, several studies have shown that sildenafil initially used for the treatment of erectile dysfunction, may have other therapeutic applications [10]–[16]. Because of its potent vasodilatory action, sildenafil has also been extensively studied for treating primary or hypoxia-induced pulmonary hypertension [11], [12], [14], [15], [17].

Endothelial dysfunction is an important defect that contributes to erectile dysfunction and vascular disease in diabetes and is associated with insulin resistance [18]. It has been reported that sildenafil can improve endothelial dysfunction after acute and chronic treatment [19]. In diabetic rats PDE-5i (SK-3530) can activate Akt-1 signaling and inhibit proapoptotic stimuli maintaining erectile function [20].

A recent report showed that chronic treatment with sildenafil improve glucose metabolism in a model of high-fat fed mice, without directly affecting Akt-1 phosphorylation [21]. The molecular mechanisms for these data still await demonstration; a possible involvement of cGMP pathway in insulin signal transduction could be hypothesized.

Since several reports have shown that endothelial dysfunction is associated with insulin resistance [22]–[24], the aim of the present study was to investigate the role of sildenafil in NO production and changes in insulin signaling in HUVECs in basal and insulin resistance conditions.

## Methods

### Materials

Human umbilical endothelial cells (HUVECs), human arterial endothelial cells (HAEC) EBM-2 and EGM-2MV media were purchased from Lonza (Walkersville, MD, USA). Human fetal corpora cavernosa smooth muscle cells (hfCC) were a kind gift of Prof. Mario Maggi. Phospho-Akt-1 (Ser-473 and Thr-308), Akt-1, phospho eNOS (Ser-1177), eNOS and iNOS antibodies were obtained from Cell Signaling (Waltham, Mass). TRIZOL, M-MLV reverse transcriptase and Platinium High Fidelity Taq were purchased from Invitrogen (Paisley, UK). BSA, Arginine, Glucose and Glucosamine (Gluc-N) were obtained from Sigma (St Louis, Mo). LY 294002, nitro-L-arginine methyl ester (L-NAME) a non selective inhibitor of all NOS isoenzymes (26) and DAF-2DA were purchased from Calbiochem (San Diego, CA). Sildenafil powder was obtained from Viagra pills (Pfizer Inc); pills were ground into powder and dissolved in deionized H_2_O. The drug solution was filtered (0.45 µm pore size) and the solution was applied to a 285 mL Sephadex G-25 (superfine) column, equilibrated before using in deionized H_2_O at 20°C [25]. Sildenafil was eluted with 500 mL of 1% formic acid, lyophilized and resuspended in deionized H_2_O, and then was used to stimulate cell cultures. The Sildenafil concentration of 1µM was chosen based on data from Luedders et al. [26].

### Cell culture

HUVEC and HAEC were growth in EGM-2MV at 37°C in an atmosphere of 95% air–5% CO_2_. HfCC were growth in M199 medium (Invitrogen) supplemented with 10% fetal bovine serum (FBS, Invitrogen) and antibiotics (50 U of penicillin and 50 µg of streptomycin/ml). HUVEC and HAEC from passages 3 to 6 were cultured for 72 hours in presence of 5.5 mmol/L glucose, 30 mmol/L glucose (high glucose; HG), 10 mmol/L Gluc-N with or without 1 µM of sildenafil (5 or 72 h) and/or 10^−7^ M insulin. LY 2940002 (30 µM) was used to block the PI3K and inhibit the PI3K/Akt pathway.

### RT-PCR analysis

The expression of endogenous PDE5 was determined by reverse transcription (RT) of total RNA followed by PCR analysis. Total RNA was extracted using TRIZOL. One microgram of total RNA was reverse transcribed using M-MLV reverse transcriptase according to the manufacturer's protocol. PCR of the cDNA was performed using Platinium High Fidelity Taq using the following pairs of primers:

1. PDE5 sense: (5′-3′) ACC GCT ATT CCC TGT TCC TT
2. PDE5 antisense: (5′-3′) AAG GTC AAG CAG CAC CTG AT

PCR parameters were: 35 cycles (94°C for 45s, 58°C for 45s, and 72°C for 1 min).

The PCR conditions were optimized for actin gene used as control.

### Real-Time Quantitative PCR

Single-stranded cDNA was synthesized from 4µg total RNA samples using High-Capacity cDNA Archive Kit (Applied Biosystems). cDNA (40 ng) was amplified by RT-PCR using ABI PRISM 9700 System and TaqMan reagents. The 20× Assays-on-demand gene expression used were: PDE-5 (Hs00903251_m1), iNOS (Hs00167248_m1), and eNOS (Hs00167166_m1). Human RNA 18s were used for samples normalization. Each reaction was carried out in triplicate.

### Western Blot Analysis

Cells were incubated for four hours in EBM-2 additioned with 0,1% BSA. After starvation cells were incubated in EGM-2MV with HG or with 10 mM Gluc-N to induce insulin resistance for 72 h with or without 1 µM sildenafil, added every 24 h. Cells were treated with 100 nM insulin during the last 30 min. of incubation. Extracts were obtained by lysing the cells in lysis buffer (50 mM Tris-HCl pH 7.6, 100 mM NaCl, 2 mM EDTA, 1 mM MgCl_2_, 1 mM CaCl_2_, 1% Triton X-100, 10% glycerol, 100 mM NaF, 1 mM phenylmethylsulfonyl fluoride, 2 mM sodium ortovanadate, 5 mM sodium pyrophosphate and protease inhibitor). Protein concentration was determined by the method of Bradford, using bovine serum albumin (BSA) as a standard. 100 µg of proteins from each lysate were separated on a 10% SDS/PAGE gel and transferred to nitrocellulose membranes. Then membranes were incubated with the following antibodies: anti-Akt-1, anti-phospho Ser-473 Akt-1, anti-eNOS, anti-phospho Ser-1177 eNOS, anti-iNOS. The primary antibody was visualized using horseradish peroxidase-conjugated anti-rabbit or anti-mouse IgG secondary antibodies and enhanced chemiluminescence.

### NO production determination

The FACScalibur (Fluorescent Activated Cell Sorting, Becton Dickinson) was used to quantify NO production. Endothelial cells were washed with PBS and starved for 1 h at 37°C in M199 phenol free medium plus 0.1% BSA. After 30 min. different inhibitors were added when required (LY 294002 30 µM, L-NAME 1 µM). Then 30 min. later sildenafil (1 µM) was supplemented when demanded. Arginine (100 µM) was incubated 1h later and after 1h diaminofluoresceine-2 diacetate (DAF-2DA, 5 µM) probe was added. After 20 min. insulin (100 nM) has been added for 1h. Finally endothelial cells were collected and analyzed with FACS, by the Cellquest program (Becton Dickinson).

### Statistical analysis of data

Data are representative of three or more independent experiments. Results are reported as mean ± SE or as percent increase respect to control where appropriate. Differences between treatments were analysed by ANOVA followed by Student's test and Mann Whitney test. A p value<0.05 was considered significant.

## Results

### PDE5 expression in HUVECs

**Figure 1:**
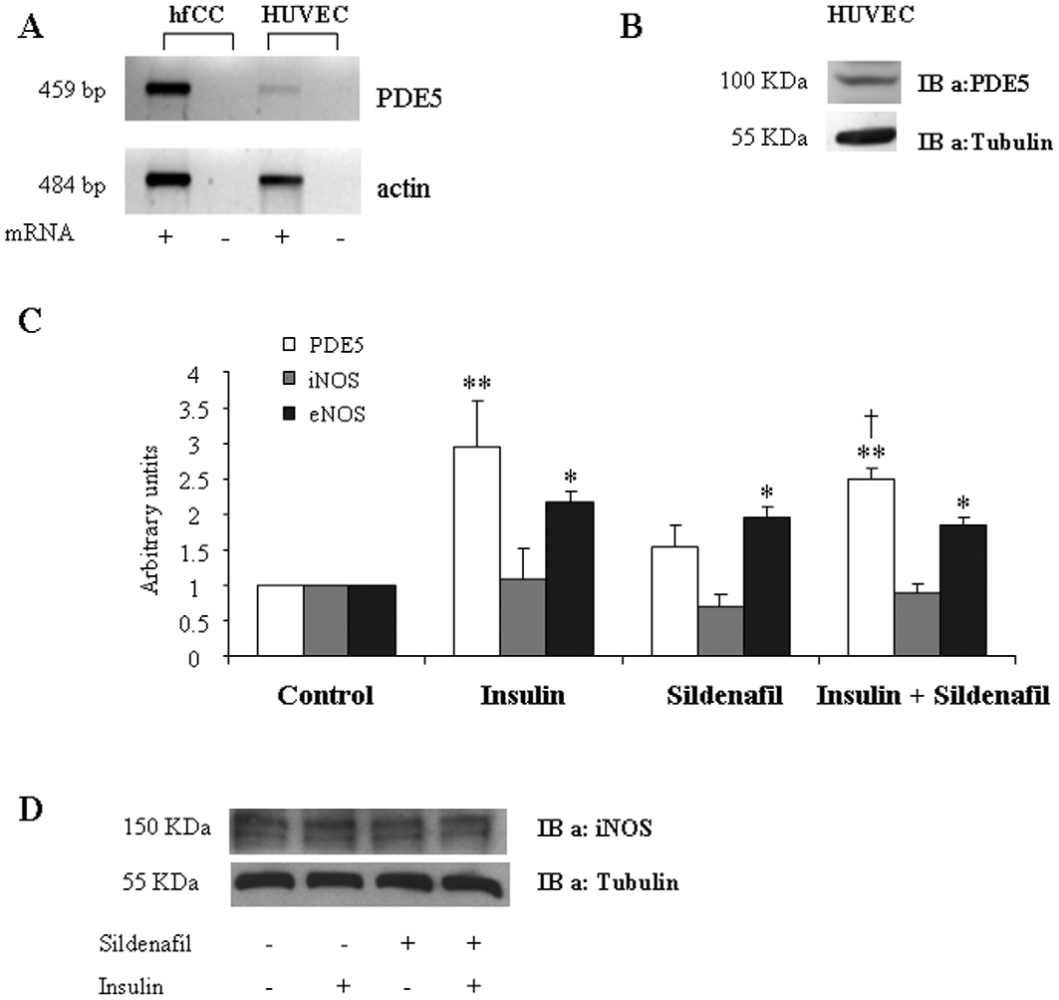
PDE5 and iNOS expression in human endothelial cells. **A**: PDE5 mRNA expression in HUVEC and hfCC cells: RT-PCR shows that HUVEC express detectable levels of PDE-5 mRNA. Actin was used as control. **B**: Western Blot analysis for PDE5 protein expression. **C**: Real time PCR shows that insulin (30 min) and/or sildenafil (5 h) treatments induce PDE5 and eNOS but not iNOS expression *p<0.05 *vs* Ctrl, **p<0.01 *vs* Ctrl; † *p*<0.05 *vs* sildenafil. **D**: A representative Western Blot shows that insulin (30 min) and/or sildenafil (5 h) treatments do not alter iNOS protein levels.

PDE-5 mRNA expression, measured in HUVECs cell line by RT-PCR, was detectable compared to the expression in human foetal corpora cavernosa (hfCC) cells used as positive control (Fig. 1A). Furthermore, Western Blot studies showed a significant expression of PDE-5 protein (Fig. 1B).

### Insulin up-regulates eNOS and PDE5, whereas sildenafil modulates only eNOS expression in HUVECs

Treatment with sildenafil (1 µM/5 h), insulin (100 nM/30 min), alone and in combination induced a 2-fold increase of eNOS but not iNOS mRNA expression, that remained roughly unchanged after treatment. iNOS protein expression was also unchanged in the same conditions (Fig. 1D). Interestingly, the positive effect of sildenafil on eNOS expression was not amplified by co-treatment with insulin. PDE-5 mRNA expression was up regulated (∼3 fold) by insulin alone or in combination with sildenafil (∼2.5 fold, Fig. 1C), whereas sildenafil alone did not significantly modify it. These data show that in our cellular model PDE5 expression is responsive to insulin treatment. Also, HUVEC cells respond to sildenafil in terms of eNOS expression in accordance with previous data, which showed that sildenafil increases eNOS expression in pulmonary vessels [27].

### Sildenafil increases Akt-1 phosphorylation in insulin-resistance conditions

**Figure 2:**
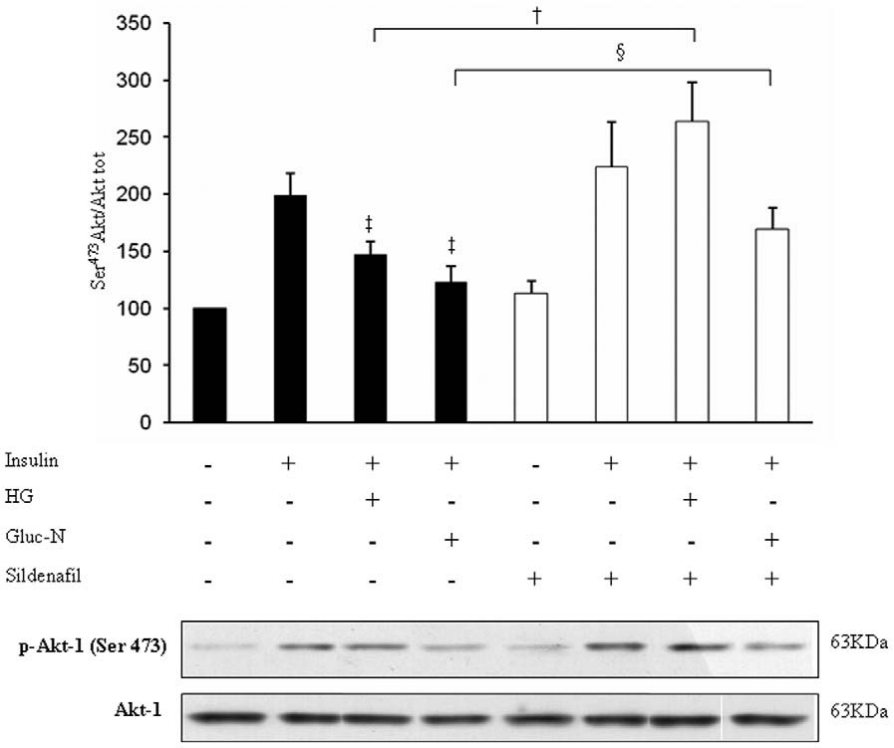
Akt-1 phosphorylation after sildenafil treatment. Insulin plus sildenafil treatment (1 µM/72 h) enhances Akt-1 phosphorylation in insulin resistance condition respect to the same conditions without sildenafil. Full bars represent conditions without sildenafil, dashed bars represent sildenafil treatment. Ctrl was expressed as 100%. Means ± S.E. (n = 5). ‡ *p*<0.05 *vs* insulin; †*p*<0.05 *vs* HG+insulin; § *p*<0.05 *vs* Gluc-N+insulin.

In order to validate our model of insulin resistance, we measured Akt-1 Ser^473^ phosphorylation in HUVECs cultured in HG and Gluc-N for 72 h. In keeping with previous observations, Akt-1 phosphorylation induced by insulin is impaired in HUVECs exposed to HG (30 mM) or Gluc-N (10 mM) [28], [29] respectively of ∼27% and ∼37% (Figure 2). Interestingly, in the same experimental conditions sildenafil co-treatment (1µM/72h) restored the ability of insulin to stimulate Akt-1 phosphorylation in presence of HG (+75% vs HG+insulin) or Gluc-N (+38% vs Gluc-N+insulin).

### Sildenafil improves eNOS Ser^1177^ phosphorylation in basal and in insulin-resistance conditions

**Figure 3:**
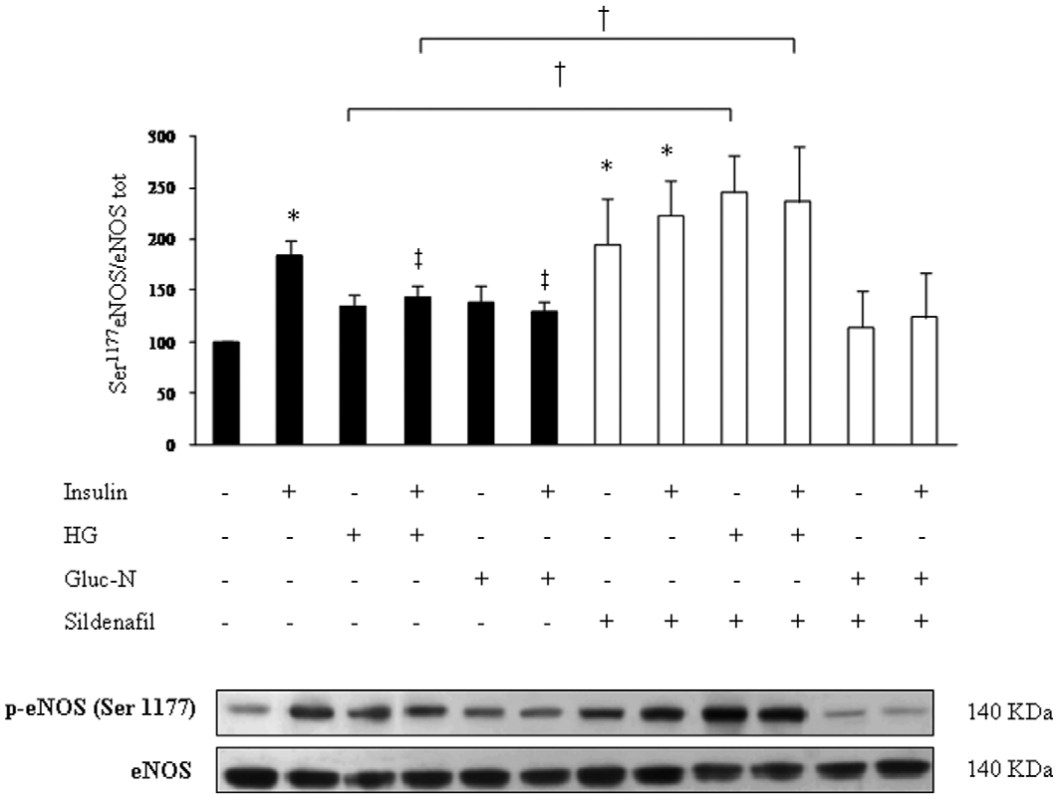
eNOS Ser^1177^ phosphorylation after sildenafil treatment. Sildenafil chronic treatment (1 µM/72 h) enhances eNOS phosphorylation in basal condition and after HG treatment, but not significantly after Gluc-N treatment. Full bars represent conditions without sildenafil, dashed bars represent sildenafil treatment. Ctrl was expressed as 100%. Means ± S.E. (n = 5). *p<0.05 *vs* Ctrl, ‡ *p*<0.05 *vs* insulin; † *p*<0.05 *vs* HG; †† *p*<0.05 *vs* HG + insulin.

**Figure 4:**
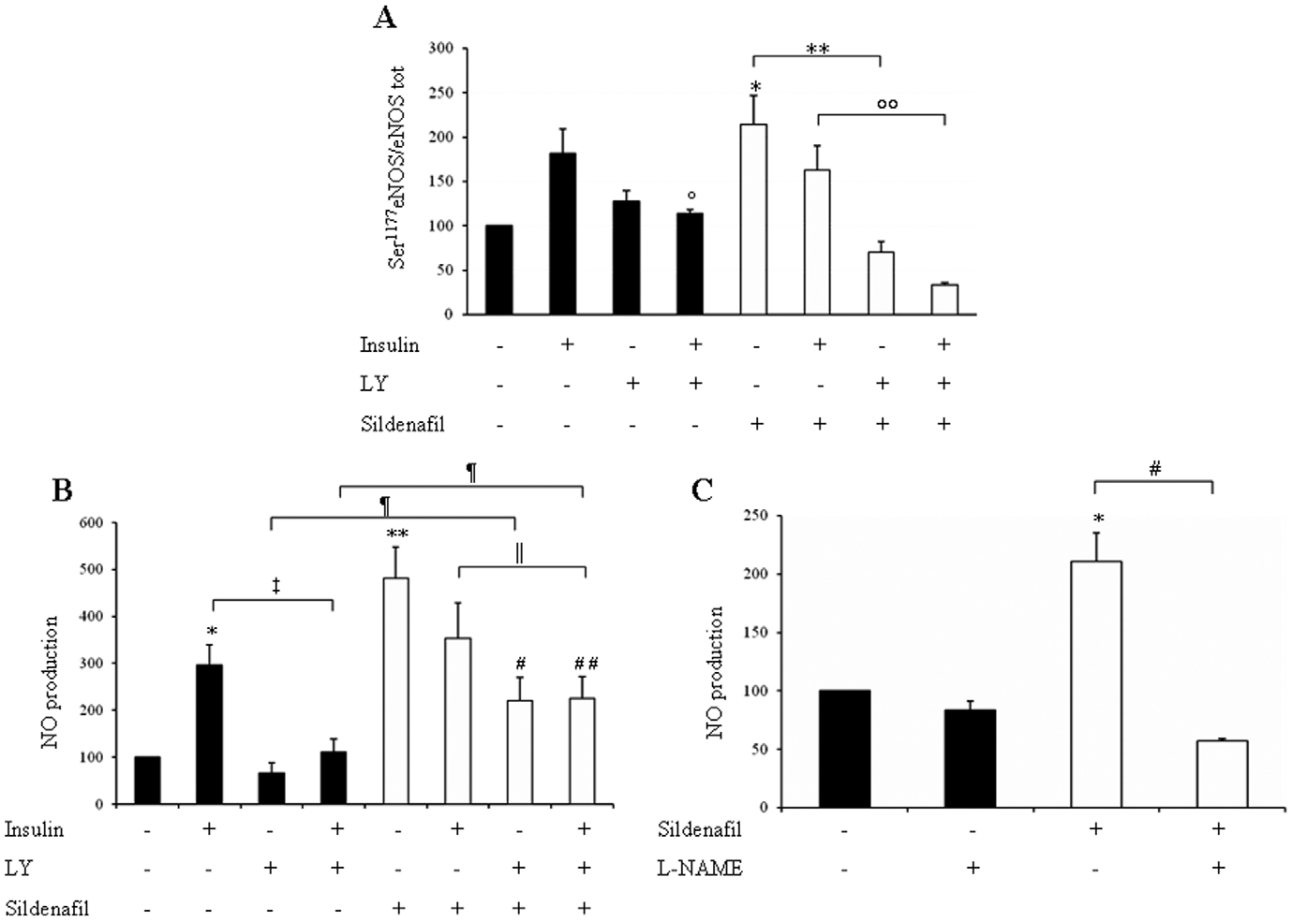
eNOS Ser^1177^ phosphorylation and NO production induced by acute treatment with sildenafil. **A**: Western Blot analysis of eNOS Ser^1177^ phosphorylation. Means ± S.E. (n = 4). * *p*<0.05 vs Ctrl; ° *p*<0.05 vs insulin; **p<0.01 vs sildenafil; °°*p*<0.05 vs sildenafil+insulin. **B**: Sildenafil treatment (1 µM/5 h) induces NO production that is partially inhibited from LY. Means ± S.E. (n = 6). *p<0.05 *vs* Ctrl; **p<0.01 *vs* Ctrl; ‡ *p*<0.05 *vs* insulin; ¶ *p*<0.05 *vs* LY; ¶¶ *p*<0.05 *vs* LY+insulin; # *p*<0.05 and ## *p*<0.01 *vs* sildenafil; ∥ *p*<0.05 *vs* sildenafil+insulin. **C**: L-NAME effects on NO production induced by sildenafil treatment. L-NAME reduces NO production induced by acute treatment with sildenafil. Means ± S.E. (n = 6). *p<0.05 *vs* Ctrl; # *p*<0.05 *vs* sildenafil. Full bar represent conditions without sildenafil, dashed bars represent sildenafil treatment.

Since eNOS is a downstream effector of Akt-1, we studied eNOS Ser^1177^ phosphorylation in the same conditions shown in figure 2. Interestingly chronic (1 µM/72 h) and acute (1µ M/5 h) sildenafil treatment enhanced eNOS Ser^1177^ phosphorylation by ∼2 and 2.2 fold respectively (Fig. 3 and Fig. 4A) (*p*<0.05) under basal conditions. Moreover eNOS phosphorylation induced by acute sildenafil treatment is inhibited by LY294002 a PI3K inhibitor (Figure 4A). In accordance with previous data [30], HG reduced insulin induced eNOS Ser^1177^ phosphorylation by 25% (*p*<0.05). Sildenafil exposure (1 µM/72 h) markedly increased eNOS phosphorylation in HG conditions, both in the absence (+83% p<0.05) and in the presence (+74%, p<0.05) of insulin co-treatment (Figure 3), differently from what observed for Akt-1 Ser^473^ phosphorylation, where sildenafil was efficient only in the presence of insulin. As expected, eNOS phosphorylation was decreased by Gluc-N, in the presence of insulin (29%, *p*<0.05) and co-treatment with sildenafil was unable to improve eNOS phosphorylation, differently to what was observed for Akt-1 phosphorylation (Figure 3).

### Treatment with sildenafil induces NO production, not exclusively via PI3K pathway

In order to confirm that the increase in eNOS Ser^1177^ phosphorylation induced by sildenafil displayed biological effects in term of NO production, we studied intracellular generation of NO by flow-cytometric analysis. Insulin (100 nM) stimulation for 1h induced a significant increase of NO production (*p*<0.05). Sildenafil treatment (1 mM/5 h) markedly enhanced NO production in HUVECs compared to control (*p*<0.01); such effect was ∼1.5-fold higher than that obtained with insulin. Co-treatment with insulin blunted the effect of sildenafil, probably because of the insulin-induced increase in PDE-5 expression, previously described in fig. 1. In order to evaluate if sildenafil effects were mediated by PI3K pathway, the same experiments were performed in the presence of LY294002, a PI3K inhibitor. LY treatment reversed insulin effect in the absence of sildenafil (*p*<0.05), as expected [31]. In the absence of insulin LY markedly blunted sildenafil-induced NO production (*p*<0.01). However, LY did not completely reverse the effect of sildenafil since NO levels were not reduced to the baseline levels. These data suggest that in our model sildenafil effects are not mediated only through the PI3K pathway (Figure 4B).

In order to confirm whether NO generation induced by sildenafil was due to eNOS activation, NO production studies were repeated in the presence of L-NAME, a NOS inhibitor. As expected, L-NAME treatment completely reversed to baseline levels both insulin- (data not shown) and sildenafil-induced NO generation (1µM/5h). (Figure 4 C). These results were confirmed also in HAEC (data not shown).

### NO production in insulin-resistance conditions in presence of sildenafil

**Figure 5:**
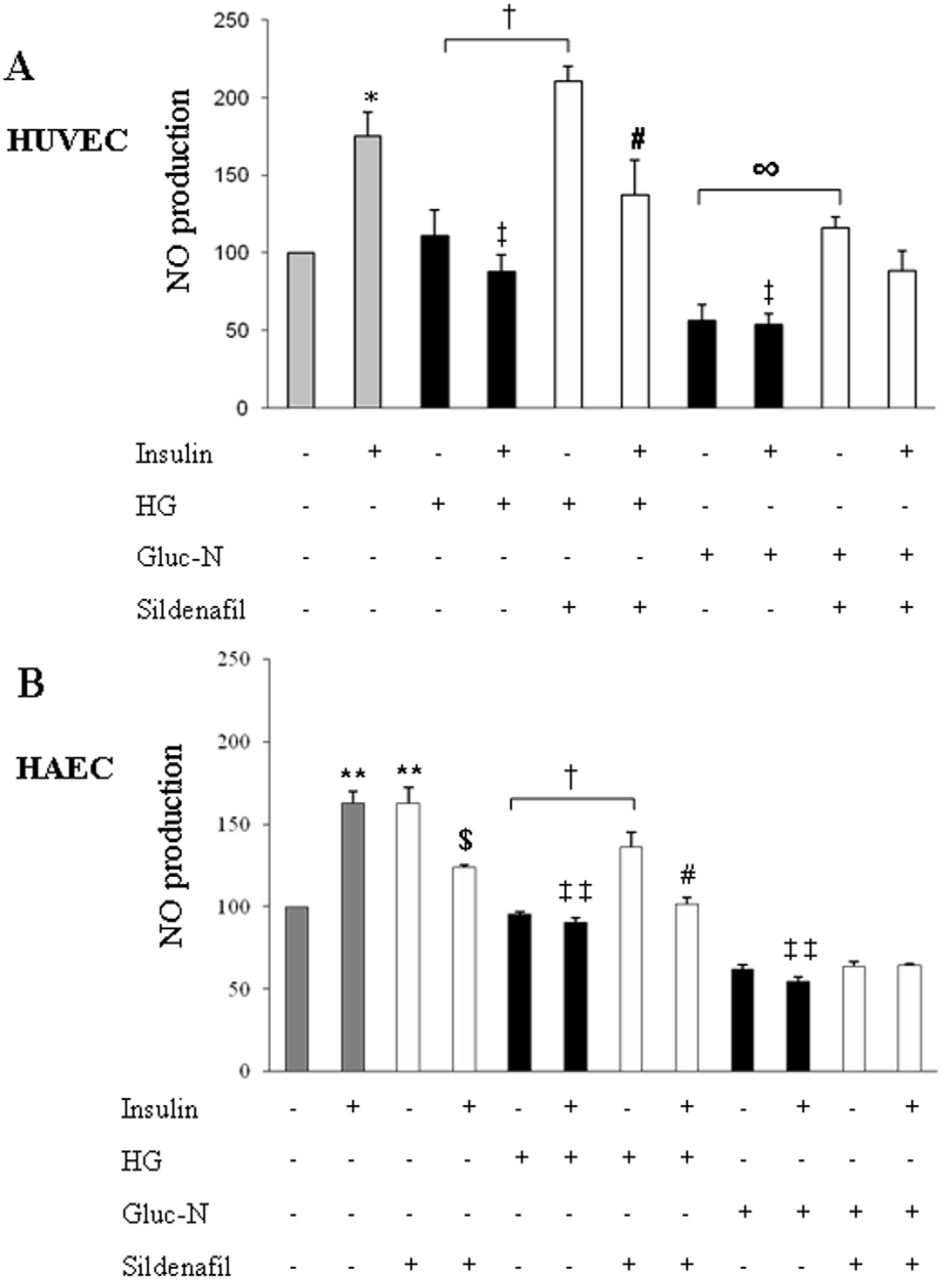
NO production in insulin-resistance conditions after sildenafil treatment. Sildenafil treatment induces NO production in insulin resistance condition (HG and Gluc-N) in HUVEC (panel A) and HAEC (panel B). Full bars represent conditions without sildenafil, dashed bars represent sildenafil treatment. Means ± S.E. (n = 6). *p<0.05 and **p<0.01 *vs* Ctrl; ‡ *p*<0.05 and ‡‡ *p*<0.01 *vs* insulin; † *p*<0.05 *vs* HG; $ *p<*0.05 *vs* sildenafil; # *p*<0.05 *vs* sildenafil+HG; **∞** *p*<0.05 *vs* Gluc-N.

As already established [7], [8], in accordance with eNOS phosphorylation studies (figure 3), conditions simulating insulin resistance (HG and Gluc-N) markedly inhibited NO production induced by insulin in HUVECs (*p*<0.05). Sildenafil treatment (1 µM/72 h) significantly increased NO generation, both in the presence of HG (*p*<0.05) and Gluc-N (*p*<0.05). Moreover insulin blunted sildenafil-induced increase in NO generation under insulin resistance conditions. Such effect was significant in HG conditions (*p*<0.05), but did not reach statistical significance with Gluc-N (Figure 5A). Similar data were obtained in HAEC (Figure 5B).

## Discussion

The present study confirms that HUVECs express PDE5 mRNA as previously reported [32] and shows for the first time that its expression is regulated by insulin. HUVECs acute treatment (5 h) with sildenafil induced a sharp increase in NO production independently of insulin stimulation. Furthermore, sildenafil was able to reverse the suppression in insulin-induced NO production, caused by the PI3-K inhibitor LY294002. Pre-treatment with L-NAME, a NOS inhibitor, completely blocks the burst of NO production induced by sildenafil, claiming for a NOS- dependent effect. Chronic treatment with sildenafil (72 h) increased NO production both in basal conditions as well as in insulin resistance conditions.

It is well known that insulin promotes vasodilation and increases blood flow by modulating eNOS activity and expression through activation of the PI3-K/Akt-1 signalling [8], [33]. As expected [9] insulin stimulation induced NO production in HUVECs and inhibition of PI3K pathway by LY294002 reduced insulin-stimulated NO production. Our results show for the first time that in endothelial cells sildenafil enhances NO production by PDE5 inhibition and eNOS activation through a PI3K dependent pathway. We hypothesize that sildenafil, through specific inhibition of PDE-5, could increase intracellular concentration of free Ca^2+^ by c-GMP. Moreover it is possible that sildenafil increases intracellular Ca^2+^ levels in a cGMP independent manner by activating K^+^ channels with large conductance (BKCa) [26]. It is known that the association of Ca^2+^/calmodulin complex with eNOS increases eNOS Ser^1177^ phosphorylation and decreases Thr^495^ dephosphorylation [34]. This mechanism could amplify NO production in response to the intracellular Ca^2+^ elevation. Our results show that sildenafil does not enhance Akt-1 phosphorylation in basal conditions, although Akt-1 Ser^473^phosphorylation has been shown to increase after sildenafil stimulation in striatal [35] and corpora cavernosa smooth muscle (20). Sildenafil differently, increases Akt-1 Ser^473^ phosphorylation in HUVECs when associated with insulin co-treatment in presence of higher oxidative stress levels (HG and Gluc-N), improving insulin action. These data are in accordance with previous studies demonstrating that sildenafil decreases ROS and nitrotyrosine cell production [36], [37]. Interestingly, in basal conditions sildenafil increased NO production without modifying Akt-1 phosphorylation (Fig. 2 and 3). Such effect was only partially reversed by PI3K inhibitor LY, without reaching baseline levels. This suggests a partial involvement of PI3K in the observed effect, whereas we can exclude the involvement of Akt-1 phosphorylation. It is possible that PI3K activates eNOS by modulating other kinases like atypical protein kinases C, p70 S6 kinase and SGK-1. These effects are insulin-independent. These data are in accordance with a work of Ayala, who found that in mice with an high fat diet sildenafil improved insulin sensitivity measured with hyperinsulinemic-euglycemic clamp with no increased levels of Akt-1 phosphorylation [21].

Hyperglycaemic impairment of nitric oxide (NO) production by endothelial cells is implicated in the effect of diabetes to increase cardiovascular disease risk. As already reported [38], [39], we found that in insulin resistance conditions, insulin treatment does not induce NO production; chronic treatment with sildenafil improves NO production in basal conditions as well as in insulin resistance conditions in e-NOS-dependent manner. This effect was reduced when insulin resistance was induced by Gluc-N. In these conditions, the positive effect of sildenafil on eNOS phosphorylation was completely inhibited by Gluc-N. However eNOS mediated NO production was partly restored even in presence of Gluc-N. These results could be explained by the different mechanism of glucotoxicity. In fact, it is known that Gluc-N, by activating hexosamine pathway, causes O-GlcNac modifications at protein sites which are involved in insulin signalling [40]. Interestingly, under insulin resistance conditions we observed that insulin blunted the increase in NO generation induced by sildenafil. We demonstrated that insulin treatment was able to up-regulate PDE5 mRNA expression in HUVEC. It is possible that insulin increased PDE-5 mRNA expression through the transcription factors Sp1, AP-1 and AP-2. Indeed, AP-1 consensus sequence has been located at – 485 from the starting PDE-5 gene expression sequence (http://variome.kobic.re.kr/SNPatPromoter/snpatpromoter.jsp?id=NM_001083).

As a consequence, it is possible that insulin might reduce intracellular cGMP through an increased activity of PDE5, with a consequent decrease in Ca^2+^/CaM dependent eNOS activation and subsequent NO production.

These findings indicate that sildenafil improves insulin signalling and NO production in endothelial cells, probably by reducing oxidative stress induced by hyperglycemia and by increasing intracellular Ca^2+^ levels.

The novel finding that insulin stimulates PDE-5 expression can explain the reduced response to sildenafil therapy in diabetic patients with elevated insulin resistance and hyperinsulinemia. Our data clarify the mechanism through which sildenafil improves insulin action in endothelial cells. Further investigation are necessary to discover all the substrates involved in this process and to understand if this novel action of sildenafil is specific for this molecule or it is a class effect of PDE-5i.

In conclusion, we hypothesize that sildenafil can be potentially used to improve insulin action in type 2 diabetic patients leading to new therapeutic strategies for type 2 diabetes and other related cardiovascular disease.

**Figure 6:**
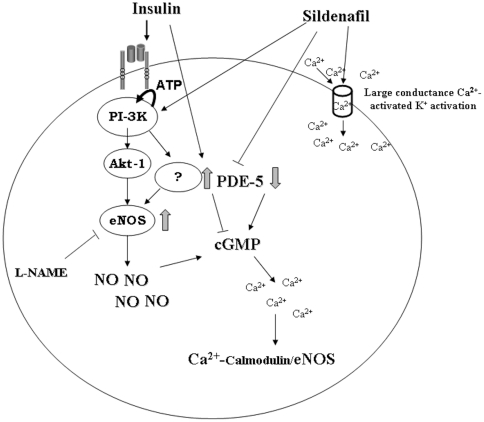
Schematic summary of sildenafil action in endothelial cells.

In Figure 6 we show a schematic summary of sildenafil action in endothelium.
